# PDS5 proteins are required for proper cohesin dynamics and participate in replication fork protection

**DOI:** 10.1074/jbc.RA119.011099

**Published:** 2019-11-22

**Authors:** Carmen Morales, Miguel Ruiz-Torres, Sara Rodríguez-Acebes, Vanesa Lafarga, Miriam Rodríguez-Corsino, Diego Megías, David A. Cisneros, Jan-Michael Peters, Juan Méndez, Ana Losada

**Affiliations:** ‡ Chromosome Dynamics Group, Spanish National Cancer Research Centre (CNIO), Melchor Fernández Almagro 3, 28029 Madrid, Spain; § DNA Replication Group, Spanish National Cancer Research Centre (CNIO), Melchor Fernández Almagro 3, 28029 Madrid, Spain; ¶ Genome Instability Group, Molecular Oncology Programme, Spanish National Cancer Research Centre (CNIO), Melchor Fernández Almagro 3, 28029 Madrid, Spain; ‖ Confocal Microscopy Unit, Biotechnology Programme, Spanish National Cancer Research Centre (CNIO), Melchor Fernández Almagro 3, 28029 Madrid, Spain; ** Research Institute for Molecular Pathology (IMP), Campus Vienna-Biocenter 1, 1030 Vienna, Austria

**Keywords:** DNA replication, cell cycle, genomic instability, BRCA2, microscopy, cohesin, fork protection, fork reversal, fork stalling, replication stress, replisome

## Abstract

Cohesin is a chromatin-bound complex that mediates sister chromatid cohesion and facilitates long-range interactions through DNA looping. How the transcription and replication machineries deal with the presence of cohesin on chromatin remains unclear. The dynamic association of cohesin with chromatin depends on WAPL cohesin release factor (WAPL) and on PDS5 cohesin-associated factor (PDS5), which exists in two versions in vertebrate cells, PDS5A and PDS5B. Using genetic deletion in mouse embryo fibroblasts and a combination of CRISPR-mediated gene editing and RNAi-mediated gene silencing in human cells, here we analyzed the consequences of PDS5 depletion for DNA replication. We found that either PDS5A or PDS5B is sufficient for proper cohesin dynamics and that their simultaneous removal increases cohesin's residence time on chromatin and slows down DNA replication. A similar phenotype was observed in WAPL-depleted cells. Cohesin down-regulation restored normal replication fork rates in PDS5-deficient cells, suggesting that chromatin-bound cohesin hinders the advance of the replisome. We further show that PDS5 proteins are required to recruit WRN helicase-interacting protein 1 (WRNIP1), RAD51 recombinase (RAD51), and BRCA2 DNA repair associated (BRCA2) to stalled forks and that in their absence, nascent DNA strands at unprotected forks are degraded by MRE11 homolog double-strand break repair nuclease (MRE11). These findings indicate that PDS5 proteins participate in replication fork protection and also provide insights into how cohesin and its regulators contribute to the response to replication stress, a common feature of cancer cells.

## Introduction

Cohesin is a ring-shaped complex that consists of SMC1, SMC3, RAD21, and SA/STAG, which can be SA1 or SA2 in somatic vertebrate cells. This ring maintains cohesion by entrapping the sister chromatids after DNA replication, which is essential for faithful chromosome segregation (1). In addition, cohesin plays an important role in genome organization, together with the architectural protein CTCF and other transcriptional regulators, that impacts gene expression and influences cellular processes such as DNA replication and DNA damage repair (2–6)

Cohesin associates with chromatin by telophase (7), and this association is dynamic (8). Loading of the complex is facilitated by a heterodimer of NIPBL and MAU2, whereas its dissociation from chromatin is mediated by the releasing factors WAPL and PDS5 (9–12) and requires opening of the SMC3-RAD21 interface (13–15). At the time of DNA replication, the replisome encounters cohesin on chromatin and it is not yet understood whether this cohesin is pushed, traversed, or dissociated and transferred behind the fork to embrace the emerging sister chromatids and establish cohesion (16–21). What is known is that a fraction of cohesin becomes stably bound to chromatin after DNA replication, which requires SMC3 acetylation and recruitment of Cell division cycle associated 5 (CDCA5), commonly known as Sororin (22–24). Binding of Sororin to PDS5 displaces WAPL, inactivating cohesin release (25). Thus, PDS5 has opposite functions depending on its binding partner: It promotes cohesin dissociation together with WAPL and stabilizes cohesive cohesin together with Sororin. There are two PDS5 proteins in vertebrate cells, PDS5A and PDS5B, whose specific functions remain ill defined (26). Knockout mice for either gene die before birth, suggesting that full compensation cannot be achieved (27). Defects in centromere cohesion are more prominent in PDS5B-deficient cells, suggesting a specific contribution of PDS5B to cohesion establishment and/or maintenance in this region (26, 27).

Cohesin promotes repair by homologous recombination (HR)^5^ in S and G_2_ phases by ensuring the proximity of sister chromatids (28, 29). In yeast, when replication fork progression is impeded by addition of hydroxyurea (HU), cohesin accumulates at replication sites and contributes to fork restart after removal of the drug (30). Moreover, under this condition, WAPL-dependent cohesin mobilization from ahead to behind stalled forks has been proposed as a mechanism that protects fork integrity (31). Whether similar mechanisms operate in mammalian cells is unknown. PDS5B has been shown to interact with BRCA2 and RAD51 during DNA replication and to be required for efficient HR-mediated DNA repair in human cells (32). *In vitro* assays further suggest that PDS5B can stimulate RAD51-mediated DNA strand invasion together with BRCA2 (33). PDS5A has not been tested in these studies.

Besides their contribution to genome maintenance through HR, several recent studies have shown that BRCA2 and RAD51 participate in replication fork protection (34). A fork remodeling mechanism known as fork reversal has been described both in prokaryotes and in eukaryotes that preserves stalled replication fork integrity (35). According to current models, stalled forks are reversed by RAD51 and DNA translocases such as SMARCAL1 (36, 37). Reversed forks become vulnerable to degradation by nucleases such as MRE11, a step antagonized by a fork protection mechanism mediated by BRCA2-dependent RAD51 filament stabilization (38–44). Additional proteins like WRNIP1 cooperate with RAD51 to safeguard fork integrity (45).

Here we have explored the contribution of PDS5A and PDS5B to cohesin dynamics in connection with DNA replication. We have found that the absence of both PDS5 proteins leads to increased stability of cohesin on chromatin and results in replication stress. Single molecule analysis indicates that fork progression rates are reduced in PDS5-deficient cells, a phenotype that can be rescued by co-depletion of cohesin or inhibition of the MRE11 nuclease. Our results also indicate that PDS5 promotes recruitment of BRCA2, RAD51, and WRNIP1 to preserve stalled fork integrity.

## Results

### Dynamic association of cohesin with DNA is altered in the absence of PDS5 proteins

**Figure 1.**
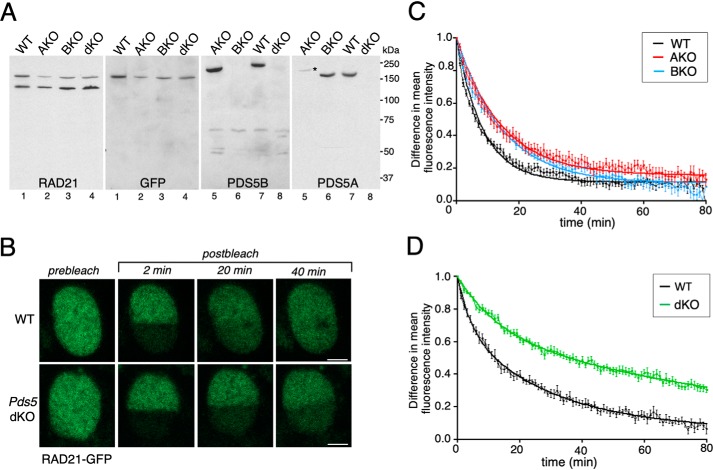
**Stabilization of cohesin on chromatin in the absence of PDS5 proteins.** *A,* immunoblot analysis of total extracts from MEFs of the indicated genotypes used in FRAP experiments. One membrane (*lanes 1–4*) was probed first with anti-RAD21 (*upper band*, RAD21-GFP; *lower band*, untagged RAD21) and next with GFP. The second membrane (*lanes 5–8*) was probed first with anti-PDS5B (*lanes 5–8*) and then with anti-PDS5A. *Asterisk* indicates remaining PDS5B signal from the first blot. AKO, *Pds5A* KO; BKO, *Pds5B* KO; dKO, *Pds5 A/B* (double) KO. *B,* images of a representative FRAP experiment comparing cohesin dynamics in WT and *Pds5* dKO MEFs. *Bar*, 5 μm. See also Fig. S1, *A* and *B*. *C,* graph plotting the difference in fluorescence intensity between bleached and unbleached regions against time (mean values and S.D.). The curve was fitted using single exponential function. WT, *n* = 13 cells; *Pds5A* KO, *n* = 13; *Pds5B* KO, *n* = 17. Cells grown in rich medium until they reached confluence were kept in low serum for 3 days before imaging. *D,* as in *C*, except that the curve was fitted using a bi-exponential function. WT, *n* = 13 cells; dKO, *n* = 12 cells. In this case, cells were kept for 5 days in low serum without (WT) or with (dKO) 4-OHT. See also Fig. S1*C*.

To address the relevance of PDS5 proteins for cohesin dynamics, we carried out inverse fluorescence recovery after photobleaching (iFRAP) analyses in mouse embryo fibroblasts (MEFs) derived from *Pds5A* KO (AKO), *Pds5B* KO (BKO), and *Pds5A/B* double KO (dKO) embryos expressing the cohesin core subunit RAD21 tagged with GFP. A CRISPR/Cas9 strategy was used to add a GFP tag to the C terminus of the *Rad21* gene to ensure physiological expression of the tagged subunit in these three cell lines (Fig. 1*A*). Single KO cell lines are homozygous for the *Pds5A* or *Pds5B* KO alleles (27). The *Pds5* dKO cell line carries conditional KO alleles of *Pds5A* and *Pds5B* in homozygosis and a *Cre-ERT2* transgene that produces a Cre recombinase that is translocated to the nucleus by addition of 4-hydroxytamoxifen (4-OHT). In the absence of treatment, this cell line behaves as WT. The GFP-tagged RAD21 protein was incorporated in cohesin complexes that were loaded on chromatin efficiently, as judged by chromatin fractionation and immunoprecipitation (Fig. S1, *A* and *B*). In iFRAP experiments, the GFP fluorescent signal was bleached with a laser in half of the cell nucleus and recovery of fluorescence was monitored by measuring the difference between the bleached and unbleached areas over time (Fig. 1*B*). We performed the experiments in quiescent cells to avoid the stable population of cohesin present in S/G_2_ cells after cohesion establishment and instead focused on the dynamic binding of cohesin to unreplicated DNA in G_0_/G_1_ cells (8, 23). To that end, confluent MEFs were cultured for 3 days in low serum before iFRAP was performed. Recovery of fluorescence after laser treatment was only mildly delayed in the PDS5A or PDS5B deficient cells (Fig. 1*C*). To test the effect of depleting both PDS5 proteins simultaneously, confluent WT MEFs were cultured in low serum in the presence of 4-OHT for 5 days, or left untreated as control (Fig. S1*C*). In *Pds5* dKO MEFs, recovery of fluorescence in the bleached region took significantly longer than in WT MEFs (Fig. 1*D*). The curve of fluorescence decay could be fitted using a double-exponential nonlinear regression and showed that cohesin complexes in PDS5-deficient cells had a chromatin residence time of 127.5 min, four times greater than the 29.6 min calculated for the slow exchange population in WT cells. These findings indicate that cohesin mobility is decreased upon elimination of the two PDS5 proteins in G_0_-arrested MEFs, as previously shown in HeLa cells (11, 12), whereas elimination of a single PDS5 protein barely alters cohesin dynamics.

### PDS5 proteins and cell cycle progression

**Figure 2.**
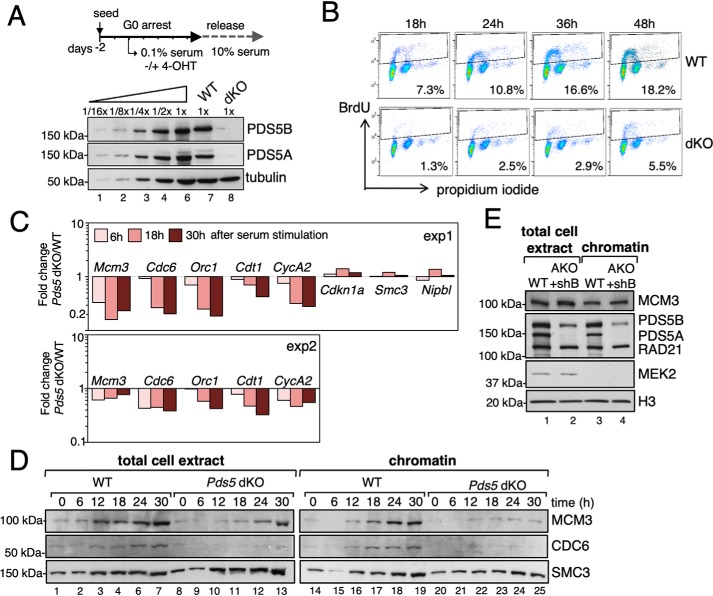
**PDS5-deficient cells show delayed re-entry into cell cycle after arrest.** *A*, scheme of the experiment and immunoblot analyses of cell extracts at the time of release into rich medium. To estimate the extent of depletion, decreasing amounts of WT cell extracts were loaded for comparison. Tubulin, loading control. *B,* BrdU incorporation profiles in WT and dKO MEFs at different time points after release from G_0_ arrest analyzed by flow cytometry. The percentage of cells that incorporated the nucleotide analogue during a 30-min pulse is indicated. *C,* quantitative PCR analyses of the mRNA levels of indicated genes. *Bars* represent the -fold change in dKO over WT cells at three time points after release. Results from two experiments are shown. *D,* immunoblot of total cell extract and chromatin fractions of WT and dKO MEFs at the indicated time points after serum stimulation. *E,* immunoblot analyses of total cell extract and chromatin fractions of G_1_ HeLa cells obtained by FACS. WT, unedited HeLa cells; AKO + shB, cells edited to knock out *PDS5A* and expressing an shRNA against *PDS5B* upon treatment with doxycycline (5 days). The cytoplasmic kinase MEK2 and histone H3 are used as controls for the fractionation procedure.

To address the impact of altered cohesin dynamics on cell cycle progression, we eliminated the two PDS5 proteins in quiescent cells and followed their re-entry into the cycle after addition of serum (Fig. 2*A*). Entry into S phase, measured by BrdU incorporation, was impaired in *Pds5* dKO MEFs (Fig. 2*B*). Initiation of DNA replication relies on licensing of pre-replication complexes (pre-RCs) that consist of the origin recognition complex, CDC6, CDT1, and minichromosome maintenance (MCM) proteins. Transcriptional reactivation of genes encoding some of these initiator proteins, as well as CYCLIN A2, is delayed in the absence of PDS5 proteins (Fig. 2*C*). This was not because of a global reduction of transcription rates, as other genes including cohesin *Smc3* or its loader *Nipbl* are transcribed at similar rates in both conditions. The delay in the synthesis of initiator proteins in *Pds5* dKO cells is likely responsible for the observed delay in assembly of pre-RC complexes on chromatin (Fig. 2*D*) and may explain delayed S phase entry upon serum stimulation (Fig. 2*B*).

We next generated HeLa cells deficient for PDS5A or PDS5B by CRISPR/Cas9 (Fig. S2, *A* and *B*). We were unable to obtain clones simultaneously deficient for both proteins, a likely indication of the relevance of PDS5 proteins for cell viability. Instead, we either transfected siRNA or expressed an shRNA against *PDS5B* in *PDS5A* KO cells to obtain cells with drastically reduced levels of both PDS5 proteins (Fig. S2, *C* and *D*). HeLa cells lacking PDS5A or PDS5B grew more slowly than control (WT) cells whereas doubly depleted cells grew very poorly (Fig. S3*A*). To monitor pre-RC assembly in the absence of PDS5 proteins in HeLa cells, these were synchronized in mitosis with nocodazole, and chromatin fractions were analyzed at different times after release into fresh media. As in MEFs, a reduction in MCM3 abundance on chromatin was observed in PDS5-deficient HeLa cells (Fig. S3*B*). However, this was most likely a consequence of the delay in recovering from the nocodazole arrest (Fig. S3*C*). When assembly of pre-RCs was assessed in G_1_ cells selected by sorting from an asynchronous population, the amount of MCM3 on chromatin was similar in cells with or without PDS5 proteins (Fig. 2*E*). Taken together, the results in MEFs and HeLa cells suggest that preventing proper cohesin release does not interfere with pre-RC complex assembly in early G_1_ but delays the synthesis of initiator proteins during exit from quiescence or from mitotic arrest. To avoid this effect, in the experiments presented below we have used asynchronous populations to address the role of PDS5 proteins on DNA replication.

**Figure 3.**
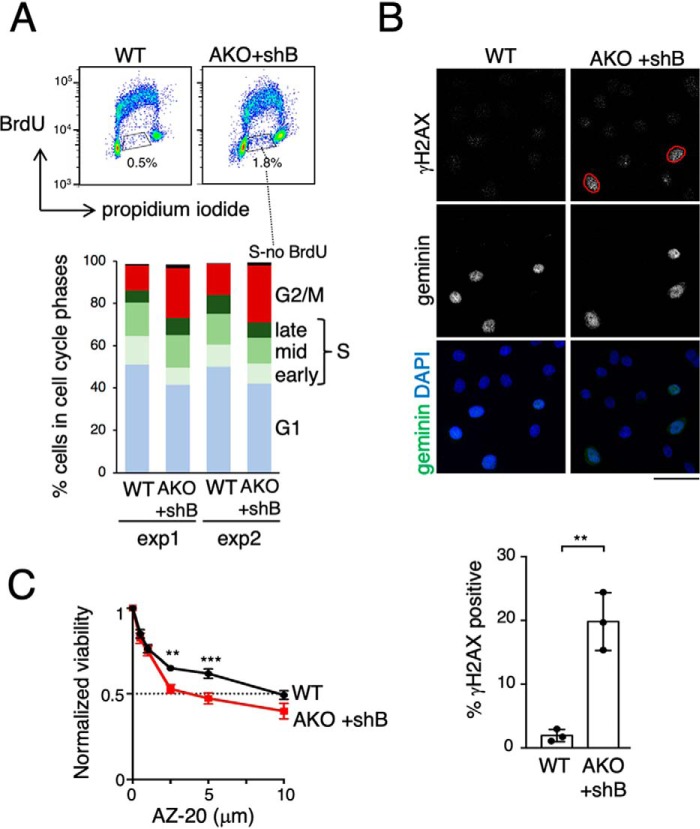
**PDS5 deficient cells show replication stress.** *A,* BrdU incorporation profiles in WT and AKO+shB HeLa cells (*top*) and histograms showing percentage of cells in each phase of the cell cycle for two different experiments (*bottom*). Cells in S phase that do not incorporate BrdU in the time of the pulse are highlighted. *B,* representative images of HeLa cells with (WT) or without PDS5 proteins (AKO+shB) stained with antibodies against γH2AX and geminin, a protein absent in G_1_. Cells showing clear γH2AX staining are indicated (*red line*). *Scale bar*, 50 μm. The fraction of γH2AX-positive cells in S/G_2_ phase (labeled with geminin) was quantified among *n* > 2000 cells in each condition and plotted below. *Bars* represent mean and S.D. from three different experiments. A *t* test was used to calculate statistical significance (**, *p* < 0.05). *C,* HeLa cells with (WT) or without PDS5 proteins (AKO+shB) were cultured in the presence of the indicated concentrations of the ATR inhibitor AZ-20 for 48 h and viability was calculated comparing with cell numbers in untreated condition. Mean and S.E. from three experiments are shown; *p* values were calculated with two-way ANOVA and Bonferroni's post-test; **, *p* = 0.0035; ***, *p* = 0.0004).

Intriguingly, analysis of cell cycle profiles revealed accumulation of HeLa cells in G_2_/M in the absence of both PDS5 proteins and also an increased fraction of cells with an intermediate DNA content between 1C and 2C that did not incorporate BrdU during the time of the pulse (Fig. 3*A*). Although the mitotic index of PDS5-deficient cell cultures was similar to that of control HeLa cells (data not shown), around 20% of cells in S and G_2_ phases lacking both PDS5 proteins displayed signs of replication stress, as evidenced by γH2AX staining (Fig. 3*B*). Moreover, these cells also show increased sensitivity to an ATM and Rad3-related (ATR) kinase inhibitor (Fig. 3*C*), which is particularly toxic in cells suffering from endogenous replication stress (46).

### Altered cohesin dynamics impair replication fork progression

**Figure 4.**
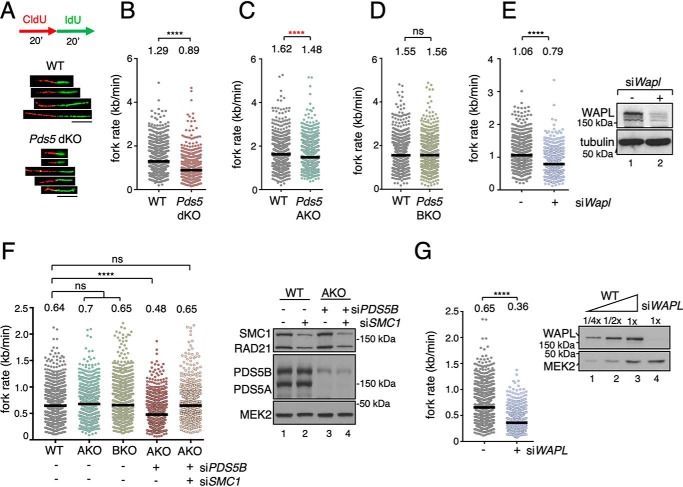
**Slow fork progression in cells lacking both PDS5 proteins or WAPL.** *A*, schematic representation of DNA fiber labeling with CldU and IdU (*top*) and examples of DNA fibers selected from micrographs of WT and *Pds5* dKO MEFs (*bottom*). *Scale bar*, 10 μm. *B–D*, dot plots showing the distribution of fork rate values in MEFs of the indicated genotypes. In *B*, WT and *Pds5* dKO pairs are the same clone treated or not with 4-OHT. *C* and *D*, WT and KO pairs are clones from littermate embryos. Results from three different clones are merged as they were not significantly different (*n* > 300 measurements per clone). Horizontal lines indicate median values (value above). *p* values were calculated with Mann-Whitney test; ****, *p* < 0.0001. *C*, *asterisk* appears in *red* because despite being statistically significant, the small difference between WT and *Pds5A* KO is unlikely to be biologically relevant. *E,* same as in *B–D*, but for two experiments in which WT MEFs were transfected with siRNA against *Wapl* (si*Wapl*) or mock transfected as control (*n* > 300 measurements per experiment per condition). The stress caused by transfection is the likely reason for the reduced fork progression rates of mock-transfected WT MEFs in these experiments compared with untransfected WT MEFs in *B–D*. Immunoblot detection of WAPL for one of the experiments is shown on the *right. F,* distribution of fork rate values for HeLa cells unedited (WT) or knockout for *PDS5A* (AKO) that were transfected with the indicated siRNAs. *n* > 900 measurements/condition pooled from three experiments. *p* values were calculated with Kruskal-Wallis test followed by Dunn's post-test; ****, *p* < 0.0001; *ns*, not significant). Immunoblot analysis for one of the three experiments is shown on the *right. G,* distribution of fork rate values in HeLa cells (WT) mock transfected or transfected with siRNA against *WAPL* after 72 h. A blot showing remaining levels of WAPL is shown on *top*. Data from three independent experiments are pooled (*n* > 900 measurements/condition; *p* values were calculated with Mann-Whitney test; ****, *p* < 0.0001).

We next addressed the cause of replication stress in PDS5-deficient cells. During DNA replication, the replisome traverses cohesin-bound genomic regions through a yet-to-be-determined mechanism. To test whether defective cohesin unloading in the absence of PDS5 proteins hinders replication fork progression, we employed single molecule analyses of stretched DNA fibers. After consecutive pulses of labeled nucleotides CldU and IdU (Fig. 4*A*, *red* and *green tracks*, respectively,), the length of the *green tracks* was measured to assess nucleotide incorporation in ongoing forks. *Pds5* dKO MEFs displayed reduced fork velocity compared with their WT counterparts (Fig. 4*B*). In the case of *Pds5A* KO MEFs, a mild (9%) reduction in median fork rate was observed (Fig. 4*C*). Despite being statistically significant, the biological relevance of small variations in fork speed should be interpreted with caution as some studies have estimated an experimental variability of around 10–15% for fork speed in fiber assays (47). No major differences were observed between *Pds5B* KO MEFs and their WT littermates (Fig. 4*D*). WAPL works together with PDS5 to dissociate cohesin (10). When *Wapl* is down-regulated in MEFs by siRNA, forks progress also more slowly (Fig. 4*E*). Overall, these results highlight the correlation between cohesin dynamics assayed by FRAP experiments and DNA replication progression assayed by fiber assays.

To corroborate our results in a different cell line, we used HeLa cells deficient for PDS5A, PDS5B, or both, and confirmed that the lack of a single PDS5 protein does not perturb fork rates whereas elimination of almost all PDS5 does (Fig. 4*F*). Down-regulation of *WAPL* in HeLa cells produces similar results (Fig. 4*G*). We reasoned that if slower fork progression in the absence of PDS5 or WAPL was due to the presence of chromatin-bound cohesin complexes hindering fork passage, the observed defects should be rescued by removing or reducing the number of such obstacles. Indeed, down-regulation of cohesin by treatment with an siRNA against *SMC1* restored fork progression in PDS5-deficient HeLa cells (Fig. 4*F*, *last column*).

### PDS5 proteins are required for BRCA2-mediated protection of stalled forks

**Figure 5.**
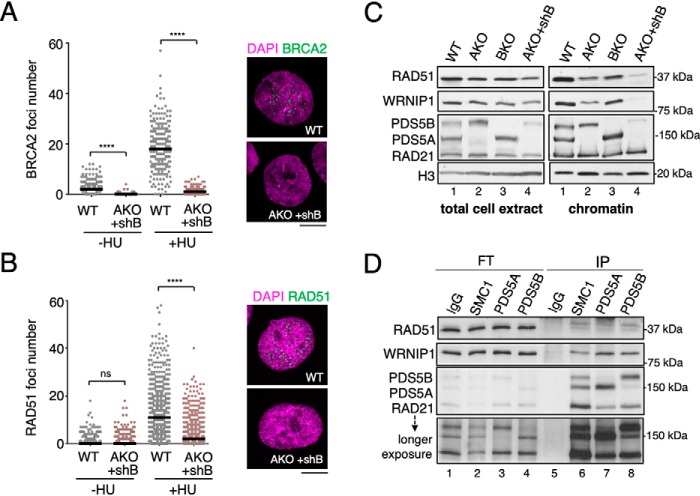
**PDS5 proteins are required for recruitment or stabilization of BRCA2, RAD51, and WRNIP1 on chromatin after HU treatment.** *A,* quantification of HU-induced BRCA2 foci in HeLa cells with (WT) or without PDS5 proteins (AKO+shB) treated with 10 mm HU for 24 h. Median values are indicated with the *black horizontal line*. At least 190 cells were analyzed per condition in each of two independent experiments with similar results (only one is shown). *p* values were calculated with Kruskal-Wallis test followed by Dunn's post-test; ****, *p* < 0.0001; *ns*, not significant. Representative images for HU-treated cells appear on the *right. Scale bar*, 10 μm. *B,* quantification of HU-induced RAD51 foci, as in *A*. At least 580 cells were analyzed per condition in each of two independent experiments with similar results (only one is shown). *C,* immunoblot analyses of total cell extracts and chromatin fractions of HeLa cells with or without one or both PDS5 proteins, as indicated, and treated with 2.5 mm HU for 24 h to analyze recruitment of RAD51 and WRNIP1. *D,* immunoblot analyses of immunoprecipitates (*IP*) obtained with antibodies against cohesin SMC1, PDS5A, PDS5B, or IgG as control, from extracts prepared from HeLa cells treated with 2.5 mm HU for 24 h under mild crosslinking condition. An aliquot of the flow-through (*FT*) was also included to estimate the efficiency of the pulldown.

When fork progression is halted, transient fork reversal followed by recruitment of fork protection proteins including BRCA2, RAD51, and WRNIP1 provides a mechanism to preserve fork integrity until the problem is solved and replication can be resumed. Previous results suggest that, at least in the context of irradiation, PDS5B promotes BRCA2 recruitment to DNA damaged foci (32). We therefore asked whether the PDS5 proteins could also participate in BRCA2 recruitment upon fork stalling to preserve fork integrity. Because normal cells display few BRCA2 foci unless subject to replication stress, we treated PDS5-proficient and -deficient HeLa cells with HU and analyzed them by immunofluorescence. Reduced numbers of BRCA2 foci were detected in cells with low levels of PDS5A and PDS5B (Fig. 5*A*). Assessment of RAD51 foci formation under the same conditions showed also a clear decrease in PDS5-deficient cells (Fig. 5*B*). In the case of WRNIP1, no suitable reagents for immunofluorescence were available and we evaluated its accumulation on chromatin upon HU treatment by immunoblot analysis of chromatin fractions. WRNIP1 association with chromatin was strongly impaired in the absence of both PDS5 proteins and, to a lesser extent, in cells carrying only PDS5A or PDS5B (Fig. 5*C*). In this biochemical fractionation assay, the accumulation of RAD51 on chromatin was also impaired, in agreement with the immunofluorescence results. Finally, a physical interaction between WRNIP1, RAD51, and PDS5 proteins was detected by immunoprecipitation with antibodies against PDS5A and PDS5B from extracts of cells treated with HU (Fig. 5*D*). An antibody against the cohesin subunit SMC1 also pulled down WRNIP1 and RAD51, suggesting that these proteins interact with cohesin bound to PDS5. Moreover, BRCA2 and RAD51 foci formation under replication stress conditions was also compromised in HeLa cells treated with an siRNA against cohesin *SMC1* (Fig. S4). Taken together, these results indicate that the two PDS5 proteins participate in the recruitment and/or stabilization of the fork protection complex at stalled forks and further suggest that they do so in the context of cohesin.

### PDS5 proteins protect nascent DNA strands from MRE11 degradation

**Figure 6.**
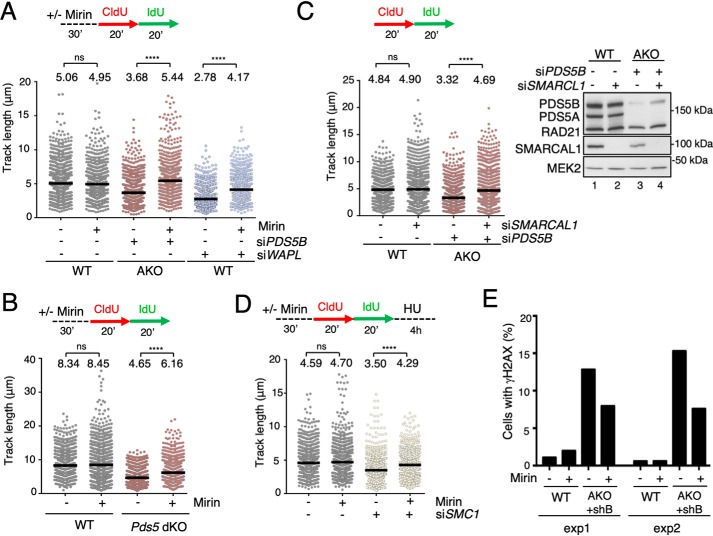
**PDS5 proteins are required to protect stalled forks from MRE11-dependent DNA degradation.** *A,* scheme of the experimental design. Dot plots show *green track* length distribution in HeLa cells unedited (WT) or knockout for *PDS5A* (AKO), mock transfected or transfected with si*PDS5B* or si*WAPL*, and treated or not with 50 μm mirin. Data from three independent experiments are pooled (*n* > 900 measurements/condition; *p* values were calculated with Kruskal-Wallis test followed by Dunn's post-test; ****, *p* < 0.0001; *ns*, not significant). In dot plots, *horizontal lines* indicate median values. *B,* as in *A*, but using WT and *Pds5* dKO MEFs. *C, green track* length was measured in HeLa cells unedited (WT) or knockout for *PDS5A* (AKO) and transfected siRNAs against *PDS5B* and *SMARCAL1*, as indicated (*left*). Protein levels were assessed by immunoblot (*right*). Data from three independent experiments are pooled (*n* > 900 measurements/condition; *p* values were calculated with Kruskal-Wallis test followed by Dunn's post-test; ****, *p* < 0.0001; *ns*, not significant). *D,* to test MRE11-dependent DNA degradation in HeLa cells with low levels of cohesin, fork stalling was induced by treatment with 6 mm HU for 4 h after CldU and IdU pulses and *green track* length measured (*n* > 900 measurements/condition; *p* values calculated with Kruskal-Wallis test followed by Dunn's post-test; ****, *p* < 0.0001; *ns*, not significant). *E,* quantification of cells showing immunofluorescent staining for γH2AX (as in Fig. 3*B*) in WT and AKO+shB HeLa cells treated or not with 50 μm mirin. Data from two separate experiments are shown.

In the absence of proper fork protection, the MRE11 nuclease degrades nascent DNAs at stalled forks. Because PDS5-deficient cells fail to accumulate BRCA2, RAD51, and WRNIP1 after HU treatment, we asked whether even in the absence of HU, stalled forks generated by chromatin-bound cohesin were susceptible to MRE11 degradation in these cells. Consistent with this possibility, DNA fiber assays revealed a significant increase in *green track* length in PDS5-deficient HeLa cells that were treated with mirin, a chemical inhibitor of MRE11, when compared with untreated cells (Fig. 6*A*). As expected, mirin had no effect on WT cells. Similar results were observed in *Pds5* dKO MEFs, although in this case the rescue was partial (Fig. 6*B*), and in HeLa cells depleted of WAPL (Fig. 6*A*, *right*). Moreover, down-regulation of the SMARCAL1 translocase involved in fork reversal restored track length in PDS5-deficient HeLa cells (Fig. 6*C*), further supporting the hypothesis that stalled forks generated by impaired cohesin dynamics are processed by fork reversal. Cells with reduced levels of cohesin exposed to HU to generate fork stalling also displayed shorter *green track* length that was rescued by addition of mirin (Fig. 6*D*). Depletion of both PDS5 proteins in cells treated with HU also resulted in an increased fraction of forks unable to resume DNA synthesis after removal of the drug, whereas depletion of a single PDS5 protein did not have consequences for fork restart (Fig. S5). Finally, we also observed that accumulation of γH2AX in PDS5-deficient cells could be partially alleviated by mirin (Fig. 6*E*). Together, these results suggest that PDS5 proteins are required to ensure proper fork protection. Moreover, cohesin mobilization mediated by PDS5 and WAPL is an essential step in this process.

## Discussion

Replication fork protection has emerged as a key mechanism to guard cells against genome instability (34, 48, 49). Under replication stress conditions, nascent DNA strands at stalled forks can be degraded by nucleases such as MRE11 unless properly protected. Many proteins participate in this protection including BRCA2, RAD51, WRNIP1, or Fanconi anemia proteins (37, 40, 43–45). We have now added cohesin and its regulator PDS5 to this list. Previous studies had established sister chromatid cohesion mediated by cohesin as an important process to ensure HR-mediated DNA repair (28, 29, 50, 51). The results presented here argue that, in addition, cohesin participates in fork protection most likely through its regulator PDS5.

Our results are consistent with those recently published by Carvajal-Maldonado *et al.* (52), who first showed that depletion of PDS5 proteins results in replication fork progression defects that are rescued by MRE11 inhibition. However, they differ in two important aspects. First, contrary to the conclusion that the role of PDS5 proteins in replication fork progression is uncoupled from BRCA2, we find that they are closely related. Second, single depletion of PDS5A or PDS5B did not result in defective fork progression or stabilization in our study, suggesting that the two variants play a redundant role in this process. In contrast, Carvajal-Maldonado *et al.* found similar defects after depleting only PDS5A, PDS5B, or WAPL. Regarding the connection between PDS5 and BRCA2, Carvajal-Maldonado *et al.* (52) argue that PDS5 binding to chromatin is independent of BRCA2, the latter being only required in replication stress conditions. Although this is true, it is important to note that the presence of unreleased cohesin in PDS5-deficient cells generates stalled forks but depletion of BRCA2 does not. Thus, BRCA2 depletion, but not PDS5 depletion, requires addition of HU to uncover a fork protection problem. The same reasoning can be used for cohesin. Reduction of cohesin levels does not cause significant problems to replication fork progression (3). However, upon replication stress caused by HU, we show here that stalled forks are not properly protected in the absence of cohesin and are degraded by MRE11 nuclease.

Regarding the apparent discrepancies in single *versus* double depletion of PDS5 proteins, our results are consistent with the observation that absence of a single PDS5 protein does not alter cohesin unloading, as measured by FRAP both in MEFs (this study) and HeLa cells (11), and by measuring the amount of cohesin present on chromatin by immunofluorescence in the two cell types (12, 27). Moreover, we show that both PDS5A and PDS5B interact with fork protection pathway components. It is conceivable that the ratio of cohesin to PDS5A/B is slightly different in HeLa cells (used in our study) and U2OS cells used by Carvajal-Maldonado *et al*. (52). Alternatively, additional factors may account for the differences in our results. Nevertheless, both studies support the model that stably chromatin-bound cohesin hinders the advance of the replication fork and that fork reversal occurs upon fork stalling in the absence of PDS5 proteins.

**Figure 7.**
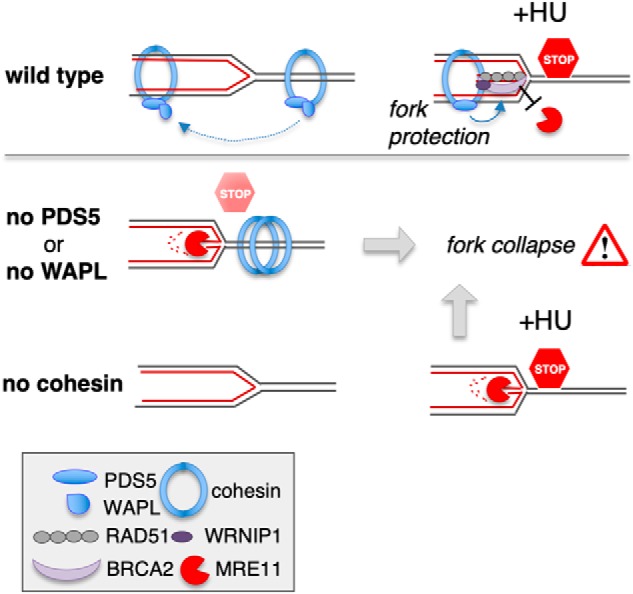
**Model for cohesin and PDS5 function at stalled forks.** *Top,* cohesin ahead of the replisome needs to be unloaded by PDS5-WAPL to allow replication fork passage. Released cohesin is reloaded in the wake of the fork (20). In the event of fork stalling caused by HU, cohesin-PDS5 reloaded behind the fork promotes the recruitment of fork protective factors RAD51, BRCA2, and WRNIP1 to stabilize stalled forks. *Middle,* in absence of PDS5 or WAPL, impaired cohesin mobilization would result in fork stalling without addition of HU. Impaired recruitment of fork protective factors would lead to MRE11 degradation of nascent DNA strand and eventually to fork collapse. *Bottom,* the absence of cohesin under replication stress conditions generated by addition of HU has similar consequences for replication forks.

The model presented in Fig. 7 proposes that PDS5 and WAPL are required for transferring cohesin from ahead to behind the fork and this has two effects. First, it allows proper progression of the replisome that would otherwise be hindered by the presence of cohesin. Second, it promotes protection of stalled forks being upstream of the BRCA2 protection pathway. The relative importance of these two effects may depend on the cell type, as for HeLa cells we find complete rescue of fiber track lengths by mirin, whereas in MEFs the rescue is only partial, suggesting that in the human cells the main effect is on the fork protection pathway, whereas in mouse cells both effects are in place. Cohesin transfer may involve a transient opening of the ring in concert with replisome components, a mechanism proposed to explain cohesion establishment during DNA replication (19, 20). When this transfer is impaired by depletion of PDS5 or WAPL, or when cohesin is absent, recruitment of fork protection proteins is reduced and the stability of the stalled fork is compromised. A requirement of WAPL-mediated cohesin mobilization to preserve stalled fork integrity has been recently reported in budding yeast grown in the presence of HU (31). We cannot conclude from our results that PDS5 proteins are directly responsible for this recruitment. However, mammalian 2-hybrid experiments and co-immunoprecipitation reactions with purified proteins showing a direct interaction between PDS5 and BRCA2 support this possibility (32, 33). PDS5 proteins have in fact emerged as critical regulators of cohesin behavior and function through their ability to interact with many regulators including WAPL, Sororin (CDCA5), ESCO1 acetyltransferase, and HASPIN (9, 25, 27, 53, 54). Their interaction with components of the fork protection pathway adds yet another activity of cohesin to ensure accurate duplication and segregation of the genome.

## Experimental procedures

### MEF isolation and culture

Three mouse lines were used in this study: *Pds5A* +/−, *Pds5B* +/−, and *Pds5A f/f; Pds5B f/f; Cre-ERT2* (27). Mice were housed in a pathogen-free animal facility following the animal care standards of the institution. All procedures have been revised and approved by the required authorities (Comunidad Autónoma de Madrid). Primary MEFs were isolated from E12.5 embryos and cultured in DMEM supplemented with 20% FBS at 37 °C under 90% humidity and 5% CO_2_. With the exception of FRAP experiments, performed in MEFs immortalized with SV40, all other experiments reported here use primary, low passage MEFs. To ablate *Pds5A* and *Pds5B* expression simultaneously, double conditional knockout MEFs (*Pds5A f/f; Pds5B f/f; Cre-ERT2*) were cultured in medium with 1 μm 4-OHT for at least 5 days and the efficiency of elimination of PDS5A and PDS5B proteins was assessed by Western blotting. For RNAi, 100 nm siRNA against *Wapl* (Table S1) was introduced in MEFs using the Neon Transfection System (Invitrogen) and knockdown efficiency was assessed after 72 h by Western blotting.

### Generation and culture of HeLa cell clones deficient for PDS5 proteins

HeLa cells were grown at 37 °C with 90% humidity and 5% CO_2_ in DMEM (Lonza) supplemented with 10% FBS (Sigma-Aldrich) and 10% penicillin/streptomycin (Invitrogen). HeLa cell clones deficient for PDS5A or PDS5B were generated with single-guide RNA (sgRNA) cloned into the BbsI site of dual Cas9/sgRNA expression vector pX458 (Addgene plasmid 48138) (55). Cells were transfected using Lipofectamine 2000 (Invitrogen) and 48 h later single GFP-positive cells were sorted into 96-well plates for isolation of single-cell clones. Following clone expansion, PDS5A and PDS5B protein levels were assessed by Western blotting to select one to be used in subsequent analyses. The region of each gene containing the sequence targeted by the sgRNAs was amplified from genomic DNA from the selected clones, cloned and sequenced to confirm gene editing. Because we could not obtain HeLa clones deficient for both PDS5A and PDS5B, double depletion was achieved by RNAi of *PDS5B* in *PDS5A* KO cells. Stable *PDS5A* KO-sh*PDS5B* and *PDS5B* KO-shB HeLa cells were generated by infection of TRIPZ lentiviral vectors carrying inducible shRNAs (V2THS_200579, V3THS_346828, V3THS_346830, Dharmacon). After puromycin selection, a polyclonal population was obtained in which expression of *PDS5B* shRNA was induced with 2 μg/ml doxycycline (Sigma) for 5 days. Alternatively, siRNA transfections for *PDS5B*, and also for *SMC1* or *WAPL* or *SMARCAL1* (Table S1), were carried out using DharmaFECT (Dharmacon) at 50 nm and cells were harvested 72 h post transfection.

### Cell extract preparation and immunoprecipitation

Whole cell extract samples for immunoblot analyses were prepared from cells lysed in Laemmli Sample Buffer at 10,000 cells/μl, sonicated, and boiled for 5 min at 95 °C. Biochemical fractionation in MEFs and HeLa cells to assess pre-RC assembly was performed as described (56), but using 0.05% Triton X-100 for cell lysis. For the analysis of WRNIP1 and RAD51 recruitment to chromatin (Fig. 5*C*), we used the protocol described in Ref. 57. MEF extract used for immunoprecipitation was prepared lysing cells on ice for 30 min in lysis buffer (0.5% Nonidet P-40 in TBS supplemented with 0.5 mm DTT, 0.1 mm PMSF and 1× complete protease inhibitor mixture (Roche)) and sonicated. Then NaCl was added to 0.3 m and the extract rotated for 30 min at 4 °C to extract chromatin-bound proteins. Salt concentration was then lowered to 0.1 m NaCl by dilution and glycerol added to 10% final concentration. Extracts were incubated with the specific antibodies for 2 h at 4 °C and rotated with 1/10 volume of protein A agarose beads for 1 h at 4 °C. For immunoprecipitation in mild crosslinking conditions (Fig. 5*D*), HeLa cells were treated with 1 mm dithiobis(succinimidyl propionate) (DSP) (Thermo Fisher Scientific) for 20 min at room temperature, and then DSP was replaced with 25 mm Tris-HCl, pH 7.5, for 10 min at room temperature to quench the reaction. Cells were scraped in ice-cold PBS, resuspended in lysis buffer (25 mm Tris-HCl, pH 7.5, 150 mm NaCl, 1 mm EDTA, 5% glycerol, 1% Nonidet P-40) and sonicated. Extracts were clarified by high-speed centrifugation and incubated with antibodies coupled to Dynabeads M-270 Epoxy (Thermo Fisher) overnight at 4 °C. Beads were washed with lysis buffer and immunoprecipitated proteins were eluted in Laemmli Sample Buffer, boiled for 5 min, and analyzed by SDS-PAGE and immunoblotting. Antibodies are listed in Table S2.

### Flow cytometry analysis

For BrdU incorporation and DNA content analysis, cells were pulsed with 10 μm BrdU (Sigma) for 30 min before harvesting. Cells were trypsinized, washed in PBS, and fixed with cold (−20 °C) 70% ethanol for at least 24 h. DNA denaturation and permeabilization was carried out with 2N HCl for 20 min at room temperature. Cells were washed twice in PBS, treated with blocking solution (1% BSA, 0.05% Tween 20 in PBS) for 15 min at room temperature and incubated with FITC-conjugated anti-BrdU antibody (BD Biosciences, Pharmingen) for 1 h at 37 °C. For DNA content analysis, cells were stained overnight at 4 °C with 25 μg/ml propidium iodide (Sigma-Aldrich) in presence of 10 μg/ml RNase A (Qiagen). For obtaining cells in G_1_ phase, cells were stained with 7.5 μg/ml Hoechst 33342 (Invitrogen) and sorted by DNA content using a BD Influx sorter (BD Biosciences).

### Single-molecule analysis of DNA replication in stretched fibers

Exponentially growing cells were sequentially pulse-labeled for 20 min with 50 μm chlorodeoxyuridine (CldU) and 250 μm iododeoxyuridine (IdU) to analyze fork progression as described (58). In some experiments, 50 μm mirin (Sigma-Aldrich) was added 30 min before CldU. To evaluate fork restart, cells were incubated in 6 mm HU for 4 h between the two pulses. In all cases, ImageJ software was used for analysis. To estimate fork rate, at least 300 tracks were measured per condition. The conversion factor 1 μm = 2.59 kb was used (59). At least 500 tracks were measured to estimate percentage of fork restart.

### FRAP in MEFs

One MEF clone of each genotype (WT, *Pds5A* KO, *Pds5B* KO, and *Pds5* dKO) was immortalized using SV40 large T antigen and used to generate RAD21-GFP expressing cell lines by CRISPR-mediated homologous recombination, as described (53). GFP expressing cells were selected by sorting. A polyclonal population was characterized by immunoblotting and immunoprecipitation (Fig. S1) and used for these studies. For *Pds5* dKO MEFs, cells at full confluence were starved in DMEM supplemented with 0.1% FBS for 5 days before performing the experiment. In constitutive *Pds5A* or *Pds5B* KO MEFs, cells were arrested for 3 days. FRAP experiments were performed in a Leica TCS-SP5 (AOBS) confocal microscope using a 40×/1.2 NA HCX PL APO objective with immersion oil. Cells were kept in a climate chamber at 37 °C with 5% CO_2_ during the experiment. Image acquisition used the HCSA software in LAS AF 2.7. Cells were photobleached with an argon laser, and the recovery was monitored by live-cell imaging taking pictures every minute. Videos were analyzed using Fiji software (60) and statistical analysis and nonlinear regression with GraphPad Prism.

### Cell staining and imaging

Cells grown on poly-L-lysine–treated coverslips were fixed for 15 min in 4% paraformaldehyde and permeabilized with 0.5% Triton X-100 in PBS for 5 min at room temperature. Coverslips were incubated with blocking solution (3% BSA, 0.05% Tween 20 in PBS) for 30 min followed by incubation with primary (Table S2) and secondary antibodies (1:200 in blocking solution) and DNA staining with DAPI (Sigma). ProLong Gold was used as mounting media. For Geminin, γH2AX, RAD51, and BRCA2 staining, images were acquired with TCS-SP5 (AOBS) confocal microscope (Leica microsystems) and analyzed with Definiens Developer XD v2.5 software (Definiens). For foci quantification, a custom ruleset was employed. In brief, nuclei segmentation was performed using DAPI signal and foci detection was performed with contrast and size splitting algorithms. The output file contains information regarding nuclei size, foci number per nucleus, size, and intensity.

### Quantitative RT-PCR

Total RNA was extracted using RNeasy kit (Qiagen) and treated with DNaseI (Ambion), and cDNAs were prepared using SuperScript II reverse transcriptase (Invitrogen). qRT-PCR analysis was performed in triplicates using the SYBR Green PCR Master Mix and an ABI Prism® 7900HT instrument (Applied Biosystems^®^). Quantifications were normalized to endogenous GAPDH, using the ΔΔ*Ct* method. Primer sequences are listed in Table S1.

### Viability assays

For treatment with the ATR inhibitor AZ-20 (AstraZeneca), 5000 cells were seeded per well in a 96-well tissue culture plate and increasing concentrations of AZ-20 added to the media. Cell viability was assessed after 48 h using a luminescent system (CellTiter-Glo, Promega), according to the manufacturer's protocol. For proliferation assays, 4000 cells (WT, *PDS5A* KO, or *PDS5B* KO) or 8000 cells (*PDS5A* KO+shB, incubated with doxycycline for 72 h before seeding) were seeded per well in 96-well tissue culture plate at day 0. Proliferation was monitored for the following 4 days using a colorimetric assay (CellTiter 96 AQueous One Solution Cell Proliferation Assay, Promega).

### Statistical analysis

Statistical analyses were performed using Prism v7.0 (GraphPad Software). For comparison of two data groups, two-tailed unpaired Student's *t* test was used if the data assumed a Gaussian distribution. Otherwise, statistical differences were assessed with nonparametric Mann-Whitney test. For multiple comparison analysis, analysis of variance (ANOVA) and Bonferroni's post-test were employed if the data assumed a Gaussian distribution. Otherwise, nonparametric Kruskal-Wallis test followed by Dunn's post-test was used. In all cases, ns, *p* > 0.05; *, *p* < 0.05; **, *p* < 0.01; ***, *p* < 0.001; ****, *p* < 0.0001.

## Author contributions

C. M., M. R.-T., V. L., J. M., and A. L. conceptualization; C. M., M. R.-T., S. R.-A., V. L., and M. R.-C. investigation; C. M., M. R.-T., S. R.-A., V. L., D. A. C., J. M., and A. L. writing-review and editing; D. M., D. A. C., and J.-M. P. methodology; D. A. C. and J.-M. P. resources; J. M. and A. L. supervision; J. M. and A. L. funding acquisition; A. L. writing-original draft; A. L. project administration.

## Supplementary Material

Supporting Information

## Footnotes

5 The abbreviations used are:

HR: homologous recombination
HU: hydroxyurea
iFRAP: inverse fluorescence recovery after photobleaching
MEF: mouse embryo fibroblasts
dKO: double KO
4-OHT: 4-hydroxytamoxifen
pre-RCs: pre-replication complexes
sgRNA: single-guide RNA
CldU: chlorodeoxyuridine
IdU: iododeoxyuridine.
