# Supplementary material for: PDS5 proteins are required for proper cohesin dynamics and participate in replication fork protection
Source: J Biol Chem. 2019 Nov 22;295(1):146–57. doi: 10.1074/jbc.RA119.011099 (PMC6952610; doi:10.1074/jbc.RA119.011099)
Supplement: Supporting Information [file supp_295_1_146__index.html]

PDS5 proteins are required for proper cohesin dynamics and participate in replication fork protection — Cohesin dynamics and DNA replication — Supporting Information 

# PDS5 proteins are required for proper cohesin dynamics and participate in replication fork protection

## Supporting Information

- Supporting Information (to be published online) - Supporting Information contains Figures S1 to S5 and Tables S1 and S2
