# Supplementary material for: PDS5 proteins are required for proper cohesin dynamics and participate in replication fork protection
Source: J Biol Chem. 2019 Nov 22;295(1):146–57. doi: 10.1074/jbc.RA119.011099 (PMC6952610; doi:10.1074/jbc.RA119.011099)
Supplement: Supporting Information [file supp_RA119.011099_155884_2_supp_432908_q1c0pt.pdf]

Supporting Information for:

# PDS5 proteins are required for proper cohesin dynamics and participate in replication fork protection

**Carmen Morales[1#], Miguel Ruiz-Torres[1#&], Sara Rodríguez-Acebes[2], Vanesa Lafarga[3], Miriam Rodríguez-Corsino[1], Diego Megías[4], David A. Cisneros[5&], Jan-Michael Peters[5], Juan Méndez[2] and Ana Losada[1*]**

Figures S1 to S5
Tables S1 to S2
Supplementary References

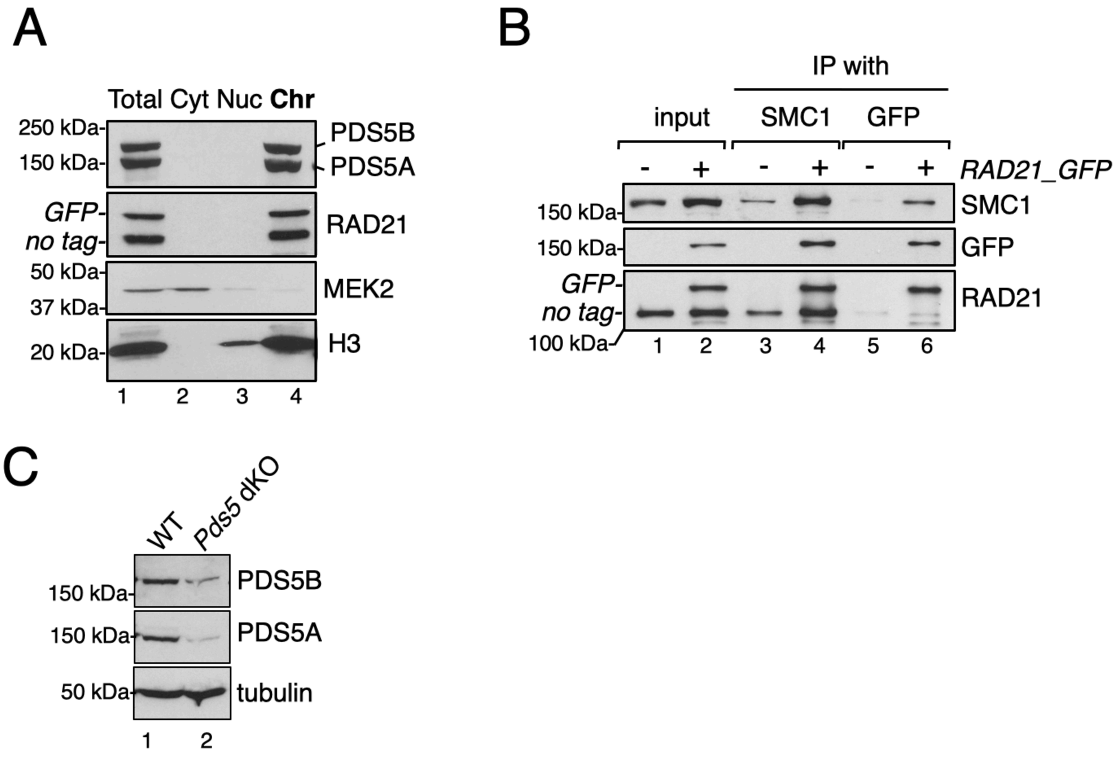


**Figure S1. RAD21-GFP protein is functional** *A,* Chromatin fractionation of asynchronously growing MEFs expressing RAD21-GFP from the endogenous locus shows normal incorporation of cohesin complexes carrying the tagged subunit into chromatin. Equivalent amounts of total cell extract (Total), cytoplasmic (Cyt), soluble nuclear (Nuc) and chromatin-enriched (Chr) fractions were analyzed by immunoblot. The cytoplasmic kinase MEK2 and histone H3 are used as controls for the fractionation procedure. *B,* Immunoprecipitation reactions with SMC1 and GFP antibodies from MEFs expressing RAD21-GFP (or not, as control) demonstrate that RAD21-GFP incorporates as well as the untagged RAD21 into cohesin complexes. *C,* Representative immunoblot of PDS5A and PDS5B elimination in *Pds5* dKO MEFs used in FRAP experiments. MEFs were seeded and after reaching confluence they were serum starved (0.1% FBS) in the absence (WT) or presence (*Pds5* dKO) of 1 μM 4-hydroxy tamoxifen (4-OHT) for 5 days before being imaged.

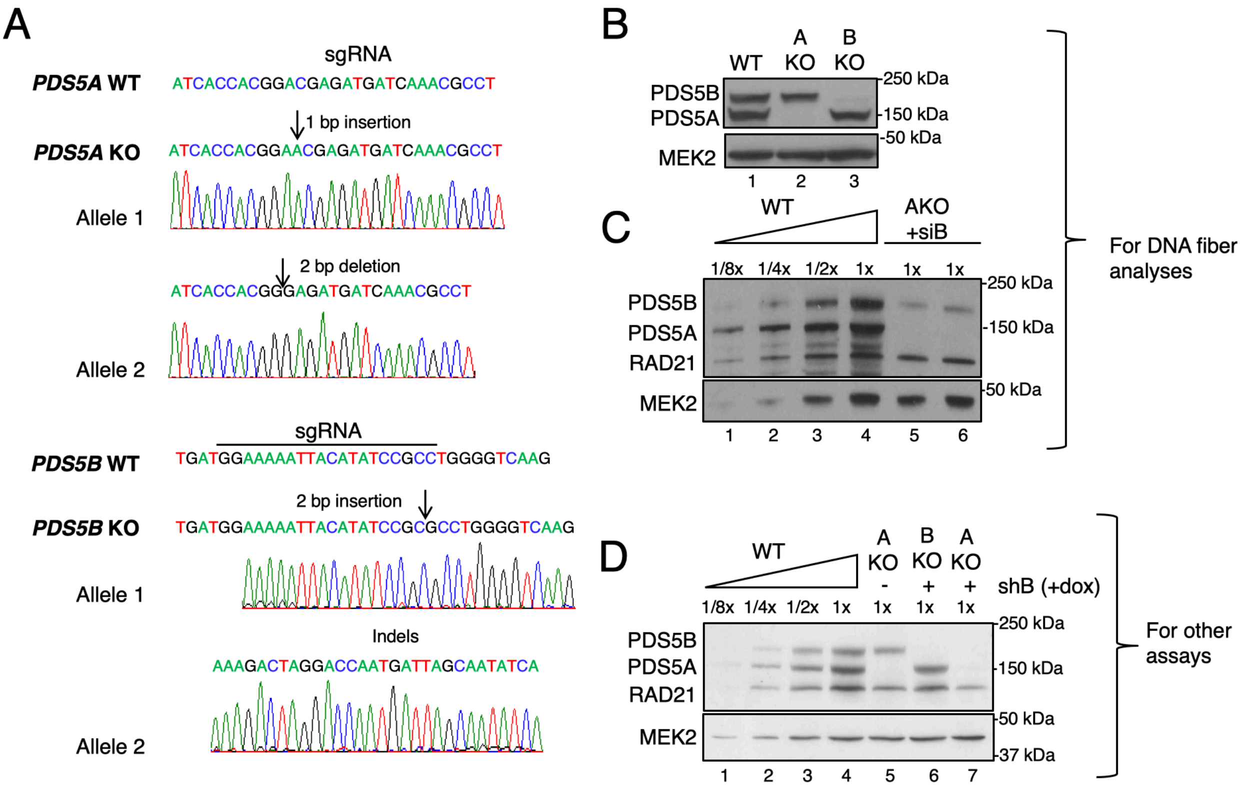


**Figure S2. Depletion of PDS5 proteins in HeLa cells.** *A,* HeLa cell clones deficient for PDS5A and PDS5B were generated by genomic editing with the CRISPR/Cas9 system. The sequence targeted by the sgRNA is indicated over the unedited allele (WT). The edited sequences identified for the clones used subsequently are also shown. *B,* Immunoblot analysis from *PDS5A* and *PDS5B* single KO clones (AKO and BKO, respectively). *C,* Representative immunoblot showing PDS5 protein levels in PDS5A KO HeLa cells treated with an siRNA against PDS5B for 72h (AKO+siPDS5B), used in fiber DNA assays. *D,* Representative immunoblot of HeLa cell lines *PDS5A* KO or *PDS5B* KO expressing an shRNA against *PDS5B* under the control of doxycycline (dox), used for other experiments. MEK2, loading control.

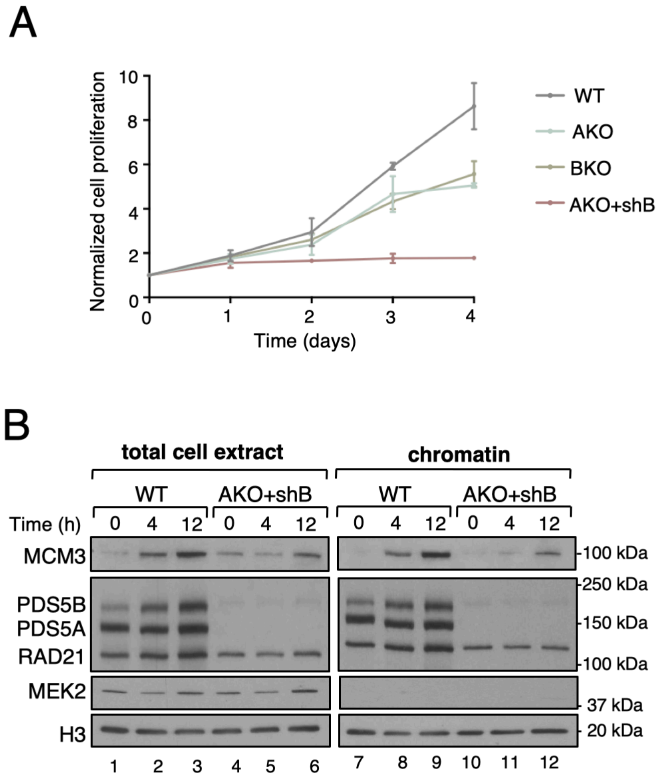


C

| | Noc release, h: | 0 | 4 | 12 |
|---|---|---|---|---|
| WT | **% G1** | nd | 49 | 40 |
| | **% S** | 7 | 8 | 33 |
| | **% G2/M** | 88 | 38 | 18 |
| AKO +shB | **% G1** | 7 | 33 | 40 |
| | **% S** | 28 | 14 | 22 |
| | **% G2/M** | 64 | 51 | 35 |

**Figure S3. Cell cycle progression defects in PDS5 deficient HeLa cells.** *A,* Proliferation rates for HeLa cells with reduced levels of PDS5 proteins. Mean and SD are represented (n=4 assays). *B,* Unedited **(**WT) HeLa cells and *PDS5A* KO HeLa cells expressing sh*PDS5B* (AKO+shB) arrested in mitosis by nocodazole were released into G1 and collected at the indicated time points to assess pre-RC complex assembly by immunoblot analyses of chromatin fractions with anti-MCM3. MEK2 (cytosolic) and H3 (chromatin-bound) are used as fractionation controls. *C,* Percentage of cells in each phase of the cell cycle at the time of sample collection after nocodazole release, as indicated by FACS.

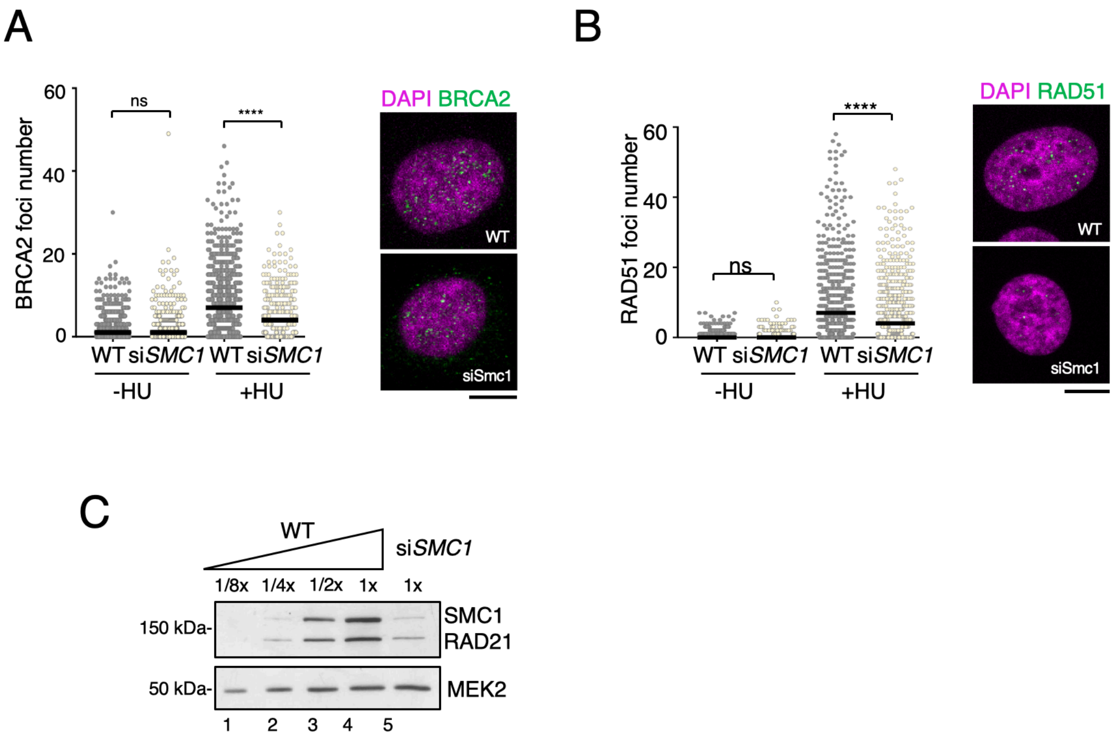


**Figure S4. Cohesin promotes BRCA2 and RAD51 foci formation after exposure to HU.** *A,* Quantification of HU-induced BRCA2 foci in HeLa cells transfected with an siRNA against *SMC1* or mock transfected, after treatment with 10 mM HU for 24 h. Median values are indicated with the black horizontal line. At least 530 cells were analyzed per condition in each of two independent experiments (only one is shown). p-values were calculated with Mann-Whitney test; ******,** $p<0.0001$; ns, no significant). Representative images are on the right. Scale bar, 10 µm. *B,* Quantification of HU-induced RAD51 foci treated as in A. At least 320 cells were analyzed per condition in each of two independent experiments. *C,* Immunoblot analysis of HeLa cells mock transfected (WT) or transfected with siRNA against *SMC1* (si*SMC1*). MEK2, loading control. To estimate the extent of depletion, decreasing amounts of WT cell extract were loaded for comparison. At least 25% (1/4) of cohesin present in WT cells remains in si*SMC1* cells after depletion, probably accounting for the milder defects observed in comparison with those in PDS5 deficient cells.

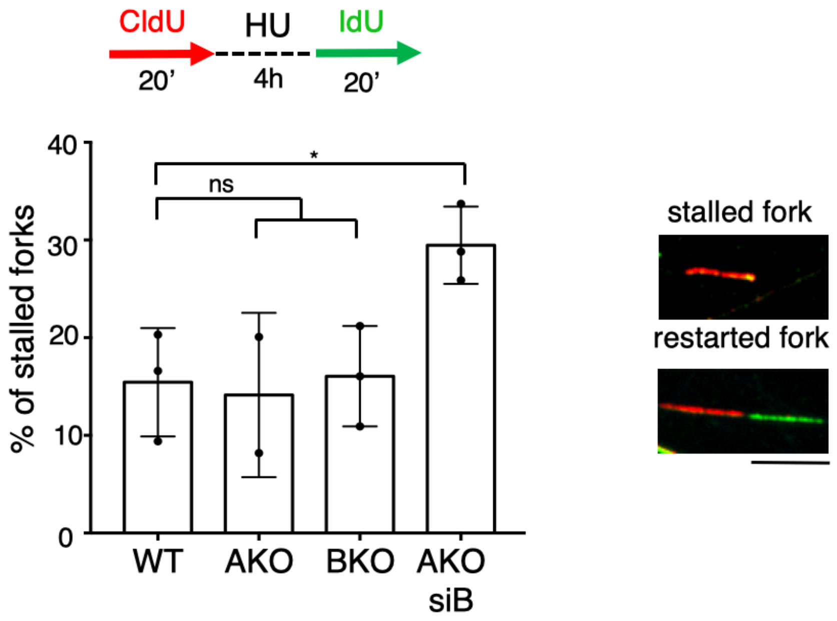


**Figure S5. Impaired fork restart in PDS5 deficient cells**. To assay fork restart in the indicated conditions, cells were labelled with CldU, treated with 6 mM HU for 4h, and pulsed with IdU after removal of the drug. The graph shows the percentage of stalled forks: Mean and standard deviation of 3 experiment (each correspond to a dot) in which >1500 measurements/condition were obtained; p-values were calculated with Anova and Bonferroni´s post test; *, $p<0.05$; ns, not significant). Representative images of stalled forks (red/CldU tracks) and restarting forks (red-green/CldU-IdU contiguous tracks) are depicted on the right. Scale bar, 10 µm.

**Table S1. CRISPR guides, primers, siRNA oligos**

| Primer name | Sequence | Use |
|---|---|---|
| sgRNA-*PDS5A* 5’-->3’ | CACCGGCGTTTGATCATCTCGTCCG | CRISPR |
| sgRNA-*PDS5A* 3’-->5’ | AAACCGGACGAGATGATCAAACGCC | CRISPR |
| sgRNA-*PDS5B* 5’-->3’ | CACCGGGAAAAATTACATATCCGCC | CRISPR |
| sgRNA-*PDS5B* 3’-->5’ | AAACGGCGGATATGTAATTTTTCCC | CRISPR |
| *PDS5A*_Fwd | AGACAGGGCCTTTTCCAGAT | PCR |
| *PDS5A*_Rev | TGCTTGAAAACCAGTAAGTTTTTG | PCR |
| *PDS5B*_Fwd | TGGGGAAATGTTCAATAGAGTG | PCR |
| *PDS5B*_Rev | AAGCTTCACCTAATTGCTGTCC | PCR |
| si*Wapl* (mouse) | siGENOME SMARTpool M-047528-01 (Dharmacon) | siRNA |
| si*WAPL* (human) | CGGACUACCCUUAGCACAA (Dharmacon) | siRNA |
| si*SMC1* (human) | siGENOME SMARTpool M-006833-00-0005 (Dharmacon) | siRNA |
| si*PDS5B* (human) | siGENOME SMARTpool M-010362-00-0005 (Dharmacon) | siRNA |
| *Gapdh*_Fwd | TGCACCACCAACTGCTTAGC | RTqPCR |
| *Gapdh*_Rev | GAGGGGCCATCCACAGTCTTC | RTqPCR |
| *Mcm3*_Fwd | TTCCTCAGCTGTGTGTGGTCTG | RTqPCR |
| *Mcm3*_Rev | TCACCACCCTAGTGGCTTTC | RTqPCR |
| *Cdc6*_Fwd | ACACACTGTTTGAGTGGCCGT | RTqPCR |
| *Cdc6*_Rev | GCTTCAAGTCTCGGCAGAATTC | RTqPCR |
| *Orc1*_Fwd | TGACTTTGAAGCGGATTAGG | RTqPCR |
| *Orc1*_Rev | GTTGGGAGGGAGGAAATAAA | RTqPCR |
| *Cdt1*_Fwd | TAGTACCCCAGATGCCAAGG | RTqPCR |
| *Cdt1*_Rev | GTAGGACAAGGCCTGGGAGA | RTqPCR |
| *CycA2*_Fwd | AGTACCTGCCTTCACTCACTCATTGCTG | RTqPCR |
| *CycA2*-Rev | TCTGGTGAAGGTCCACAAGACAAG | RTqPCR |
| *Cdkn1a*_fwd | CTAGGGGAATTGGAGTCAGGC | RTqPCR |
| *Cdkn1a* _Rev | AACAGGTCGGACATCACCAG | RTqPCR |
| *Smc3*_fwd | ATTGGTGCCAAAAAGGATCA | RTqPCR |
| *Smc3*_Rev | TGAGAATCTGGTGCCGTTGC | RTqPCR |
| *Nipbl*_fwd | AGTCCATATGCCCCACAGAG | RTqPCR |
| *Nipbl*_Rev | ACCGGCAACAATAGGACTTG | RTqPCR |

**Table S2. Antibodies**

| Antibody | Use | Source |
|---|---|---|
| Rb anti-PDS5B | WB; IP | Custom made against synthetic peptide (hPDS5B aa1226-1249) at Innovagen AB |
| Rb anti-PDS5A | WB; IP | Custom made, (27) |
| Rb anti-SMC1 | WB; IP | Custom made, (61) |
| Rb anti-SMC3 | WB; IP | Custom made, (61) |
| Rb anti-RAD21 | WB | Custom made, (27) |
| Rt anti-WAPL | WB | Custom made against recombinant fragment (hWAPL aa838-1190) at CNIO Monoclonal Antibodies Unit |
| Ms anti-GFP | WB; IP | Roche, 11814460001 |
| Rb anti-MCM3 | WB | Custom made, (62) |
| Ms anti-CDC6 | WB | Millipore, 05-550 |
| Ms anti-MEK2 | WB | BD, 610236 |
| Rb anti-H3 | WB | Abcam, ab1791 |
| Rb anti-WRNIP1 | WB | Novus Biologicals, NB110-61626 |
| Ms anti-γH2AX | WB; IF | Millipore, 05-636 |
| Rb anti-RAD51 | WB | Santa Cruz, sc-8349 |
| Ms anti-RAD51 | IF | GeneTex, GTX70230 |
| Ms anti-BRCA2 | IF | Calbiochem, OP95 |
| Rb anti-Geminin | IF | Santa Cruz, sc-13015 |
| FITC-Ms anti-BrdU | FACS | BD, 556028 |
| Rt anti-BrdU (CldU) | IF | Abcam, ab6326 |
| Ms anti-BrdU (IdU) | IF | BD, 347580 |
| Ms anti-ssDNA | IF | Millipore, MAB3034 |

Rb, rabbit polyclonal; Ms, mouse monoclonal; Rt, rat monoclonal
WB, western blot; IP, immunoprecipitation; IF, immunofluorescence; FACS, Fluorescence activated cell sorting

**Supplementary References**


61. Remeseiro, S., Cuadrado, A., Gómez-López, G., Pisano, D. G., and Losada, A. (2012) A unique role of cohesin-SA1 in gene regulation and development. *EMBO J.* **31**, 2090–2102

62. Alvarez, S., Díaz, M., Flach, J., Rodriguez-Acebes, S., López-Contreras, A. J., Martínez, D., Cañamero, M., Fernández-Capetillo, O., Isern, J., Passegué, E., and Méndez, J. (2015) Replication stress caused by low MCM expression limits fetal erythropoiesis and hematopoietic stem cell functionality. *Nat. Commun.* **6**, 8548
